# Physico-chemical properties of an innovative gluten-free, low-carbohydrate and high protein-bread enriched with pea protein powder

**DOI:** 10.1038/s41598-021-93834-0

**Published:** 2021-07-14

**Authors:** Monika Wójcik, Renata Różyło, Regine Schönlechner, Mary Violet Berger

**Affiliations:** 1 Department of Food Engineering and Machines, University of Life Sciences in Lublin, 28 Głęboka St., 20-612 Lublin, Poland; 2 Department of Food Science and Technology, Institute of Food Technology, BOKU- University of Natural Resources and Life Sciences, Muthgasse 18, 1190 Vienna, Austria

**Keywords:** Nutrition, Biomaterials - proteins, Chemical engineering

## Abstract

The study aimed to determine the effect of pea protein powder on the pasting behavior and physico-chemical properties including the composition of amino and fatty acids of gluten-free bread with low-carbohydrate content. The control bread recipe was based on buckwheat flour (50 g) and flaxseed flour (50 g) as main flours. Additionally, the improving additives for this control bread such as psyllium husk (4 g), potato fiber (2 g), and guar gum (2 g) were used. The mixture of base flour was supplemented with the addition of pea protein powder (PPP) in the amount ranging from 5 to 25%. The results of Visco analyzes measured by RVA apparatus showed that the addition of 10% PPP to the control bread did not significantly differentiate peak viscosity and pasting temperature which was at the level 3115 cP and 3149 cP and 50 °C, respectively. Supplementation of low-carbohydrate bread with 10% of PPP was acceptable and significantly increased the content of all analyzed amino acids, as well as the amount of α-linolenic acid concerning the control bread. The lowest value of chemical score was observed for leucine. The EAAI (essential amino acid index) value increased from 34 to 40 when the optimal protein supplement was added. The developed gluten-free, low-carbohydrate, and high protein bread was characterized by contents of carbohydrate of 16.9%, protein of 17.1%, fiber of 13.7%, fat of 3.3% and its calorific value was 194 kcal/100 g.

## Introduction

Bread and flour-based foods are an important part of the diet of most people around the world. These products provide energy, protein, and minerals^1,2^. Recently, consumers are looking for functional breads^3^, among them gluten-free, high-protein, or low-carbohydrate bread. Although gluten-free bread recipes have been improved increasingly^4,5^, only a little research has been done on high-protein or even less on low-carbohydrate bread^6^. Low-carbohydrate bread can be proposed for people suffering from diabetes.

Concerning high protein bread, wheat bread was mainly enriched with high-protein flour from legumes. Such flours are often characterized by a high content of protein, fat, vitamins, fiber, and usually lower content of carbohydrates than wheat flour^7,8^. Also, these flours are characterized by a high content of lysine, and also improve the balance of essential amino acids in baked products^9^. Coda et al*.*^10^ studied the effects of the substitution of wheat flour with faba bean flour (30%) on the properties of obtained bread. These authors obtained an improvement in the quality of the bread protein through faba bean sourdough addition. In addition to those mentioned lupin flour was incorporated into wheat-based bread by Villarino et al*.*^8^ and another study at producing white wheat bread with increased protein, fiber, resistant starch, and decreased carbohydrate contents by partially substituting wheat flour with soy protein isolate, oat bran, and chickpea flour^6^. A low carbohydrate bread formula was also prepared using hard red spring wheat flour, soy protein, and vital gluten^11^.

An increasing part of the human population is intolerant to gluten, including the storage proteins found in wheat, rye, and barley. Therefore, scientists are looking for alternative cereals^12^. Most gluten-free raw materials are characterized by a low protein content, which affects the nutritional value of bread. Some authors studied the possibilities of substituting gluten-free bread with chickpea flour, pea isolate, carob germ flour, or soy flour^13^. According to these authors, chickpea bread had the best physico-chemical characteristics, and therefore could be a good alternative to soy proteins. In other studies, chickpea protein together with tiger nut flours was proposed as alternatives to emulsifier, and shortening in gluten-free bread^14^.

As mentioned above, there have been only a few attempts to create low carbohydrate bread, and they were mainly based on wheat flour. There are no clear reports where gluten-free bread with reduced carbohydrates, and increased protein content was studied. The aim of the study was therefore to determine the effect of varying protein contents after pea powder addition on the pasting behavior, and properties of a low-carbohydrate bread. Besides, the amino acid composition, and fatty acid content in an optimized bread were measured.

## Materials and methods

### Materials

In the present study, the following raw materials were used to make the control dough: buckwheat flour (Helcom, Poland), flaxseed flour (Bio Planet, Poland), psyllium husk (Dimica, Slovakia), potato fiber (Spiegel Hauer, Germany), guar gum (NatVita, Poland). All raw materials were purchased from a health food store. Dried yeast (Saf Instant, France), and Himalayan salt (Intenson, Poland) were also added. Also, the following high-protein ingredient was used: pea protein powder (Bio Planet, Poland) with protein contents of 78%.

### Determination of basic chemical compositions of materials and bread

The chemical compositions of flours (buckwheat, and flaxseed), and pea protein powder, such as protein content^15^, fat content^16^, ash content^17^, moisture content^18^and dietary fiber content^19^ were investigated. Carbohydrates were calculated by subtraction of protein, fat, moisture, and dietary fiber. The calorific value (per 100 g of bread) was calculated according to Costantini et al*.*^20^ using Atwater coefficients.

### Bread-making procedure

The control bread dough consisted of buckwheat flour (50 g), and flaxseed flour (50 g), psyllium husk (4 g), potato fiber (2 g), guar gum (2 g), salt (2 g), yeast (1 g), and tap water (130 ml). Buckwheat flour, and flaxseed flour were basic flour (100%), and were used in equal proportions (1:1) in the amount of 50 g each flour. Other additives were treated as technological improvers, and were additionally added to 100 g of base flour (according to baking practice—the amount of flour is given as 100%, and the ratio of the other components are converted to the weight of flour). The addition of pea protein powder (PPP) was used in the range of 5–25% as a substitute for the base flour. For example, if 10% protein was added, the percentage of buckwheat flour, and the same flaxseed flour was 45% or 45 g, together 90% of the base flour, and 10% of the added pea flour. The addition of water was the same in all the analyzed samples. Raw materials with a low carbohydrate content were selected for the basic bread recipe. The recipe composition has been selected as a result of numerous laboratory baking to obtain a good quality bread, without crumbling (disintegrate due to the lack of gluten). The bread was made according to the straight dough method earlier had been used for gluten-free bread (Ziemichód et al*.*^27^) with slight modifications. All dry ingredients were combined with water. The dough was mixed to optimum development (6 min) in a laboratory spiral mixer type GM-2 (Sadkiewicz Instruments, Bydgoszcz, Poland), and was then divided into 120 g pieces, gently rounded, and then transferred into loaf tins (95 × 60 mm top; 80 × 50 mm bottom; 40 mm deep). Fermentation was performed at 30 °C, and 80% relative humidity for 60 min, afterward the bread was baked at 210 °C for 35 min. The obtained bread was cooled to room temperature, packed in polyethylene bags, and stored for 24 h until analysis. The bread baking experiments were done in three replicates.

### Analysis of pasting properties of flour mixtures

A Rapid Visco Analyzer (RVA-4500, PerkinElmer, USA) was used to analyze the pasting properties of the control sample (C), which was the same mixture of flours (buckwheat, flaxseed), and improvers (psyllium husk, potato fiber, guar gum) as those used in baking, and described above. This mixture with varying pea protein (PPP) supplementation (5%, 10%, 15%, 20%, and 25%) was also tested the same as baking. Samples with different PPP additives were named 5CP, 10CP, 15CP, 20CP, and 25CP. On the other hand, we were also interested in examining the characteristics of buckwheat flour (BW) itself, and the mixture (BF) of buckwheat flour, and flax flour (1:1). These flours themselves did not give good quality bread, but studies of their properties are lacking in the available literature. It was useful to further explain the overlapping relationships. The measurements were made according to the approved method 22-08 (AACCI, 2000)^22^. In the beginning, we prepared flour mixtures, then we tested their moisture content, which was in the range from 11.1% to 13.7%. Based on this moisture content, the program calculated the appropriate weight of the mixture corresponding to 3.5 g of flour with 14% moisture. After weighing a sample was transferred directly into a metal RVA canister, and filled with 25 ml of distilled water. Samples (stirred at the speed of 160 rpm) were heated from the temperature of 50 °C to 95 °C for 5.5 min, maintained at 95 °C for 5 min, cooled to 50 °C in 5 min, and kept at this temperature for 5 min. The RVA software was used to evaluate the curve characteristics (Thermocline for Windows v2.2, Newport Scientific Pty. Warriewood NSW, Australia). Paste viscosity parameters recorded were peak viscosity (cP); trough (cP); final viscosity (cP), and pasting temperature (°C). The tests were replicated thrice.

### Basic properties of bread

The volume of low-carbohydrate bread was measured 24 h after baking using the millet seeds displacement method^22^. Values were calculated for 100 g of bread. The pH of the crumb of bread was tested using the pH meter 206-ph2 (Testo, Pruszków, Poland). The baking loss was calculated by measuring the weight of the dough piece before baking, and weight after baking.

### Colour measurements of bread

The colour change of the bread crumb as a result of the addition of pea protein was assessed using a colorimeter CR30-16 (Precise Color Reader, 4Wave, Tychy, Poland). The measurement was based on the CIE L*a*b* system where L* defines lightness from 0–100 (black to white), a* denotes red( +)/green(−)value, and b* the blue (−)/yellow ( +) coordinate. Three replicates of each bread sample were analyzed.

### Texture profile analysis of bread

The analysis of the texture parameters 24, and 48 h after baking was performed using the ZWICK Z020/TN2S (Zwick Roell Group, Ulm, Germany) strength testing machine with a round measuring head with a diameter of 25 mm. Bread crumb slices were cut directly before the measurement (15 mm of thickness) using a square cutter (20 mm × 20 mm). The samples were subjected to double compression to 60% of their thickness at speed of 20 mm s^-1^, which allowed the calculation of texture parameters such as hardness, springiness, cohesiveness, and chewiness^23^. The analysis was conducted in eight replicates.

### Sensory evaluation of bread

The sensory analysis of the obtained bread was carried out 24 h after baking by seventy panelists (18–70 years, 40 females, and 30 males). For the tests, square-shaped samples with dimensions of 20 × 20 mm were prepared, which were cut with a special cutter from a slice of bread of 1 cm thickness. They were then coded, and submitted for evaluation in a closed odor-free room. The following quality indicators were assessed: taste, colour, texture, odor, and overall acceptability. The degrees of liking for the low-carbohydrate bread were based on a seven-point hedonic scale (1: dislike very much, 4: neither like nor dislike, 7: like very much)^24^.

### Amino acid composition of bread

The amino acid composition was determined after the execution of protein hydrolysis. The acid hydrolysis was performed according to Davis and Thomas^25^. The hydrolysis procedure according to Schramm et al*.*^26^ was used to determine the sulfur amino acids, and tryptophan. The content of the amino acids with tryptophan was measured using the acid analyzer AAA 400 (Ingos, Prague, Czech Republic) following the methodology described by Ziemichód et al*.*^27^. Additionally, the chemical score (CS) of essential amino acids, and the EAAI index were calculated^28^.

### Fatty acid composition of bread

Gas chromatography was used to determine the qualitative, and quantitative composition of the mixture of fatty acid methyl esters (FAME) in the sample of bread prepared by ISO 12966-2:2017-05^29^. Chromatographic separation was performed using Varian 450-GC gas chromatograph with Galaxie Chromatography Data System software.

### Statistical analyses

Statistical analysis of the final results was carried out in Statistica 12.0 considering a significance level α = 0.05. Analysis of variance (ANOVA) was performed, and Tukey’s test was used to compare the mean values.

### Human participants

Authors declare that research involving human research participants have been performed in accordance with the Declaration of Helsinki. Peoples were informed and informed consent was obtained from the patients. All experimental protocols were approved by a University of Life Science in Lublin institutional committee.

### Experimental research on plants/seeds

The collection of plant material complied with relevant institutional (University of Life Science in Lublin), national (Poland), and international guidelines and legislation.

## Results and discussion

### Basic chemical composition of flours

Buckwheat flour, and flaxseed flour used to produce low-carbohydrate bread contained, respectively: 13.0 ± 0.05%, and 40 ± 0.22% protein, 3.1 ± 0.07%, and 8.8 ± 0.02% fat, 63.1 ± 0.1%, and 3.9 ± 0.07% carbohydrates, 4.1 ± 0.2%, and 34.0 ± 2.1 fiber, and 1.20 ± 0.02%, and 6.9 ± 0.4% ash content. Pea protein powder (PPP) was characterized by protein content of 78.4 ± 0.41%, carbohydrates content of 7.2 ± 0.3%, and fat content of 6.8 ± 0.09%. Buckwheat, and flaxseed flour were selected for the bread recipe based on the available literature, and chemical analysis as raw materials with a low carbohydrate content. For example, white wheat flour usually contains more than 70% carbohydrates^30^, and rice flour at most 80%^22,30^, while corn flour has usually even more than 80% carbohydrates^30^.

### Pasting behavior of flours and bread mixtures

**Figure 1:**
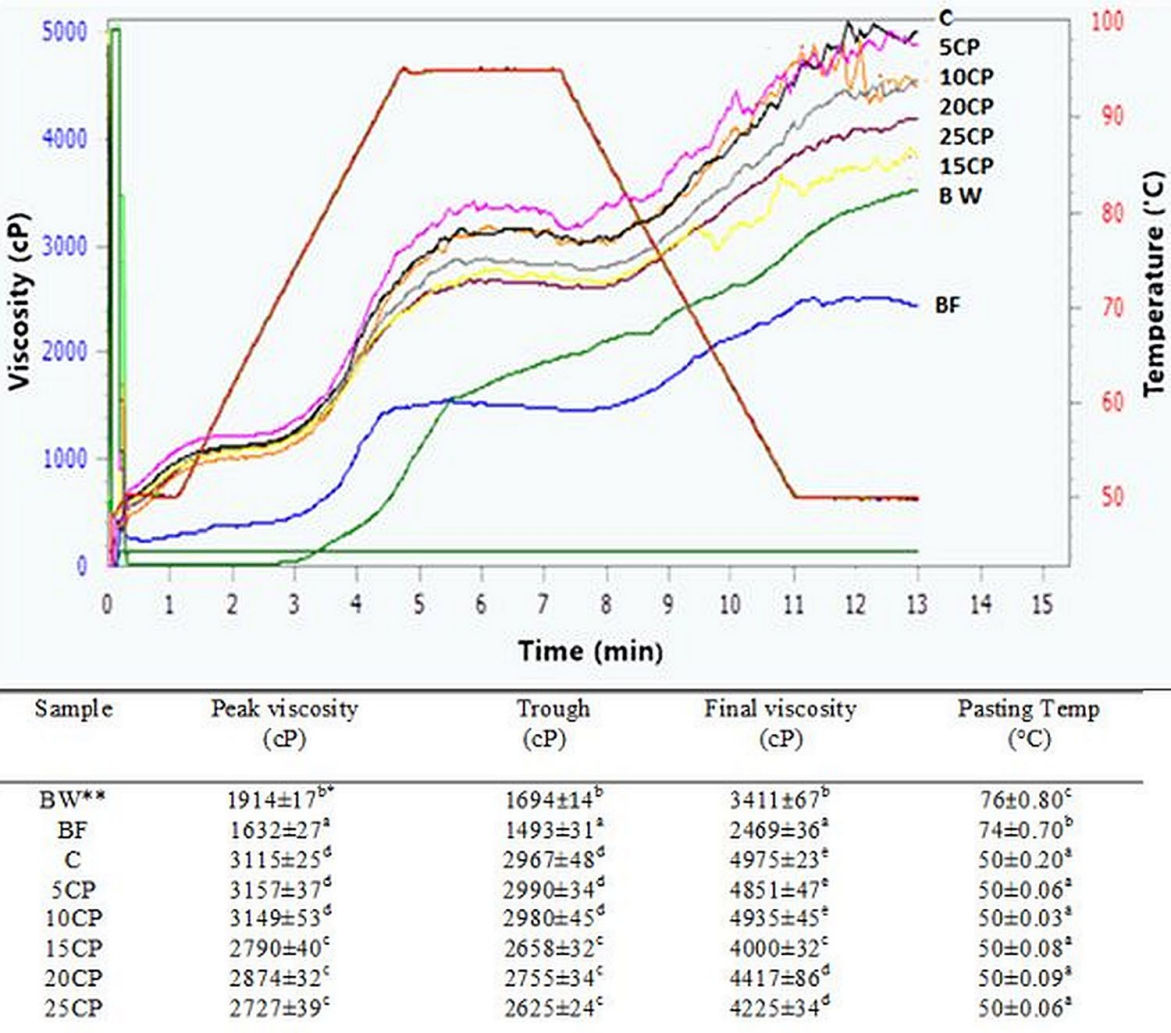
Pasting properties of studied flours, and blends with different concentration of pea protein powder: BW—buckwheat, BF—blend of buckwheat, and flaxseed flours; C—control sample, 5CP—blend of flours with 5% pea protein powder added, 10CP—blend of flours with 10% pea protein powder added, 15CP—blend of flours with 15% pea protein powder added, 20CP—blend of flours with 20% pea protein powder added, 25CP—blend of flours with 25% pea protein powder added*.* *Values in the same column marked with different letters are significantly (α = 0.05) different.

The pasting behavior of buckwheat, blend of flaxseed, and buckwheat flours, and control bread mixture with a different percentage of the PPP is shown in Fig. 1. The RVA curves obtained from the measurements are also presented.

The control sample with the addition of psyllium husk, potato fiber, and guar gum (C) had a significantly higher peak viscosity, and lower pasting temperature compared to the blend of buckwheat, and flaxseed flours (BF). The control bread (C) recipe included technological enhancers which resulted in a significant improvement in these parameters, and the addition of protein had no negative effect. As reported by Casas et al.^35^ the apparent viscosity of guar gum solutions increased with guar gum concentration, and they showed pseudoplastic behavior. In another study by Harasztos et al*.*^36^ addition of arabinoxylans, the major components of dietary fiber wheat flours showed a constant increase in all measured viscosity parameters. The authors assumed that arabinoxylans had a significant impact on viscosity despite their low concentration. The effect of dietary fiber concentration on RVA rheological properties of wheat starch/fiber systems was evaluated by Yildiz et al*.*^37^, and authors showed that peak, trough, break down, final, and setback viscosity increased; however, pasting temperature decreased as fiber concentration increased. The same results were found in our study, using a mixture of guar gum, potato, and psyllium fiber, peak, and final viscosity increased, pasting temperature decreased.

The addition of protein did not change the pasting temperature, which for these mixtures was at 50 °C. The highest viscosity values were observed in the control sample (C) with 5%, and 10% of PPP, but a PPP addition of 15%, and more resulted again in a significant reduction in the viscometric parameters. It is known that differences in the protein composition can affect pasting viscosity, as found for example by Ragaee et al*.*^34^. Xie et al*.*^38^ explained that this decrease in paste viscosity was probably due to hydrolysis of the protein rather than the starch components. Although in our studies it may have been caused by starch dilution.

In the case of buckwheat flour alone or a mixture of buckwheat flour, and flaxseed flour, the peak viscosity values were much lower, and the temperature was higher than control bread. Also, the results showed a decrease in peak viscosity by adding flaxseed flour to buckwheat flour, which could be mainly attributed to the change of carbohydrate (starch) content in the final blend. In our study flaxseed flour used in a mixture with buckwheat flour was characterized by a very low content of carbohydrates, which significantly reduced the content of the resulting blend, thus reduced its peak viscosity. Similar to our study the final viscosity of a barley-flaxseed composite blend (1:1) presented by Inglett et al*.*^31^ was lower than that of barley flour, and other composites. As these authors explained it may be due to the low viscosity contributed by the ground flaxseeds. As reported by Kaushal et al*.*^32^, flaxseed flour due to higher protein content, showed a lower swelling ability because of stronger bonding in this flour, which directly influences the peak viscosity of its blend. In other studies^33^, the addition of potato starch to wheat flour increased the peak, and final viscosity in the mixtures of wheat flour with potato starches. Also, Ragaee and Abdel-Aal^34^ explained that the high content of starch in wheat flours compared to wholegrain meals may contribute, to some extent, to the higher pasting viscosity. The peak viscosities increased significantly with an increase in the starch content in the mixtures.

### Physical properties of low carbohydrate bread with pea protein

The addition of PPP caused significant changes in the basic properties of the resulting bread (Table1). With the increase of protein, bread moisture increased from 53.4% in the control sample to 57.2% in the bread with 25% of PPP. It was also observed that bread with 5%, and 10% of PPP had a lower baking loss (23%), while at higher PPP amounts it increased again. On bread volume, PPP addition had a negative impact, a decrease was noticed. Ziobro et al*.*^38^ reported that the volume of the bread baked with pea protein was smaller than control bread. Kamaljit et al*.*^40^ noticed the same trend in the case of pea flour addition to wheat bread. The addition of PPP resulted in a slight but significant increase in the pH value of the bread crumb.

**Table 1:** Effect of different levels of pea protein powder (PPP) on the basic properties, and crumb color values of low-carbohydrate bread.

Amount of PPP (%)	Moisture of bread (%)	Baking loss (%)	Volume of 100 g of bread (cm^3^)	pH (−)
0	53.4 ± 0.52^a^*	24.7 ± 0.45^b^	296.2 ± 2.4^a^	5.3 ± 0.03^a^
5	54.8 ± 0.34^a^	23.1 ± 0.48^a^	253.3 ± 1.5^b^	5.3 ± 0.03^a^
10	55.1 ± 0.23^b^	23.6 ± 0.33^a^	225.3 ± 2.6^c^	5.4 ± 0.02^b^
15	55.4 ± 0.33^b^	28.1 ± 0.49^c^	198.6 ± 1.4^d^	5.5 ± 0.06^b^
20	56.4 ± 0.44^b^	28.4 ± 0.46^c^	182.3 ± 1.6^e^	5.5 ± 0.06^b^
25	57.2 ± 0.46^c^	28.5 ± 0.38^c^	180.5 ± 2.5^e^	5.5 ± 0.04^b^

Color values	L*	a*	b*	∆E
0	39.1 ± 0.28^a^*	6.3 ± 0.04^a^	20.6 ± 0.33^a^	
5	39.0 ± 0.39^a^	6.2 ± 0.04^a^	20.5 ± 0.15^a^	0.21 ± 0.07
10	38.9 ± 0.32^a^	6.1 ± 0.03^a^	20.7 ± 0.02^a^	0.67 ± 0.02
15	41.0 ± 0.35^b^	4.9 ± 0.03^b^	19.3 ± 0.16^b^	2.54 ± 0.23
20	40.4 ± 0.27^b^	5.1 ± 0.12^b^	20.3 ± 0.27^a^	2.14 ± 0.06
25	40.6 ± 0.23^b^	4.6 ± 0.05^b^	19.5 ± 0.05^b^	3.12 ± 0.03

*Values for the same parameters in the same kolumn marked with different letters are significantly (α = 0.05) different.

Colour parameters analysis showed that the addition of PPP up to 10% did not cause any significant change in the colour of the crumb of the low-carbohydrate bread (Table 3). Only a higher addition caused a slight but significant increase in lightness(L*), and a decrease in the a*-, and b*-value.

**Figure 2:**
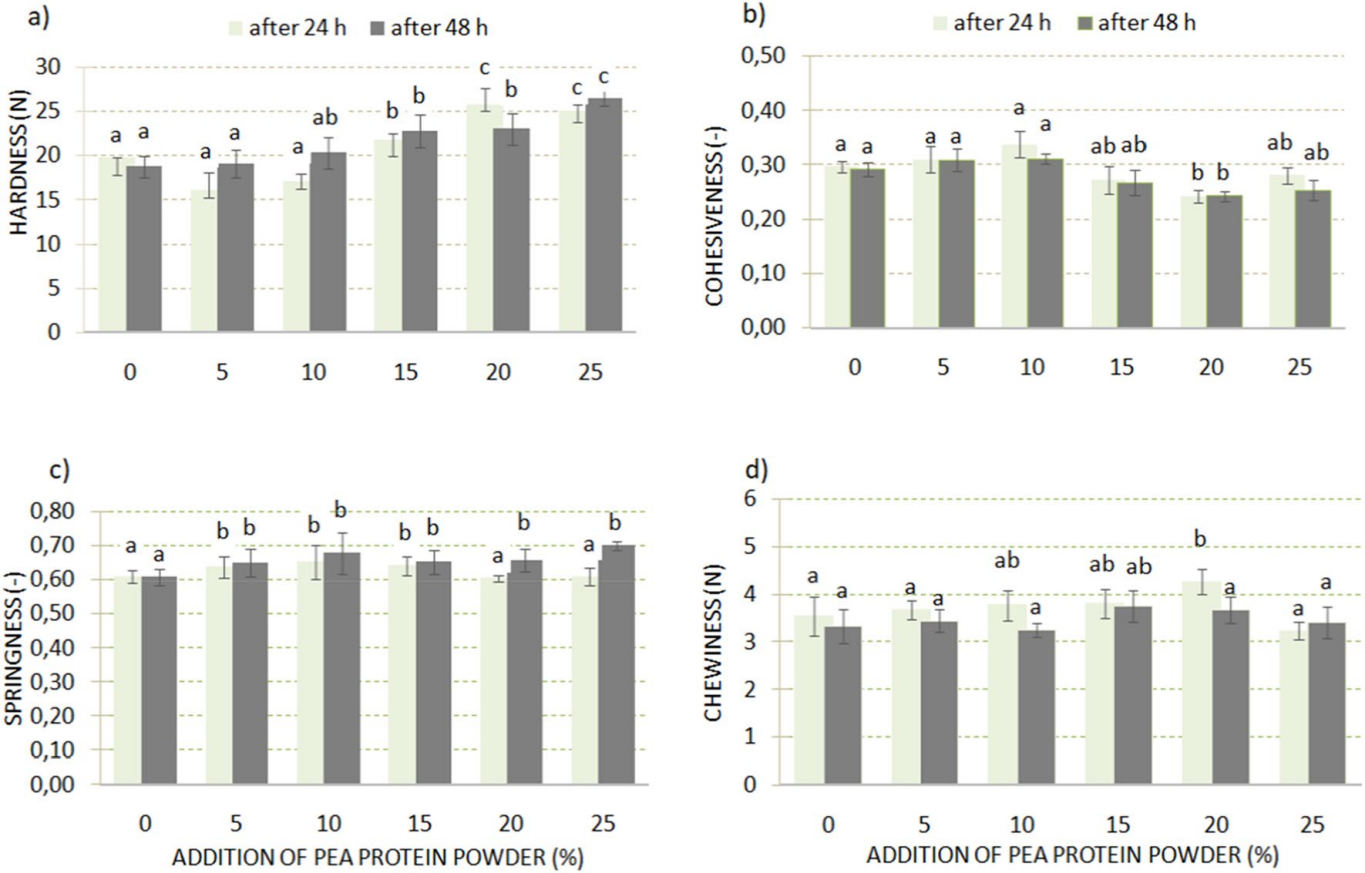
Changes of textural crumb properties of low-carbohydrate bread as a result of PPP addition: (**a**) hardness, (**b**) cohesiveness, (**c**) springiness, (**d**)chewiness; mean values in the same figure marked with different letters are significantly (α = 0.05) different.

Figure 2 presents the textural parameters of the bread crumb supplemented with PPP after 24 h, and 48 h of storage. It was noticed that the amount of PPP up to 10% did not cause changes in hardness, cohesiveness, and chewiness of bread crumbs (Fig. 2a,b,d), even after a long storage time. Only the crumb of the sample with 20% PPP addition was characterized by lower hardness after 48 h of storage compared to the sample stored for 24 h (decrease of about 2 N). However, in general, a higher share of the PPP brought about an increase in the bread crumb hardness, and chewiness, and a decrease in cohesivness. In contrast, the springiness of bread crumb (Fig. 2c) increased already with small amounts of PPP addition, as compared to control bread.

### Sensory evaluation of low-carbohydrate bread with pea protein

**Figure 3:**
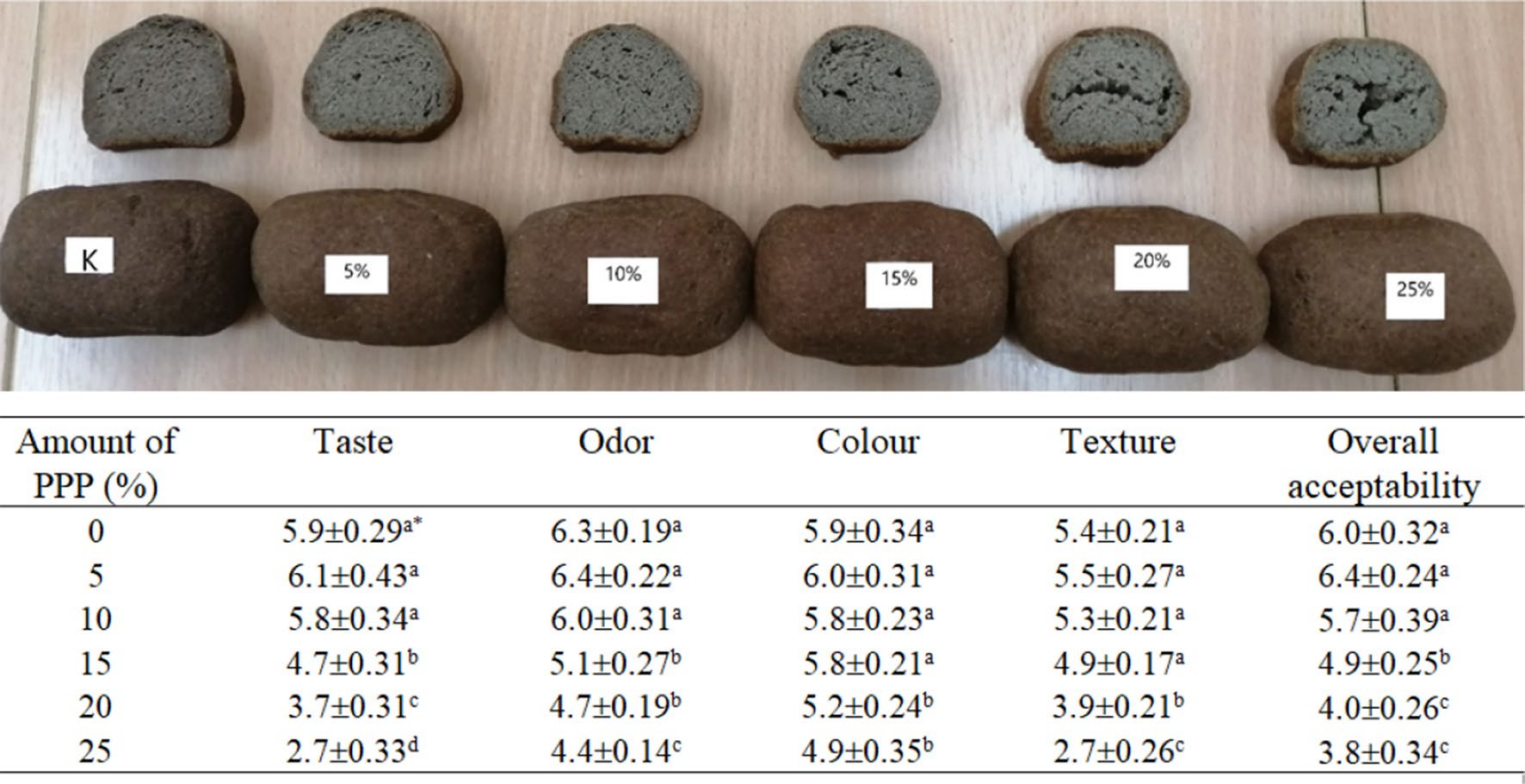
Overall view, and results of sensory evaluation of low-carbohydrate bread with the addition of 5% -25% pea protein powder (PPP)*.* The author of the photo in this figure is Monika Wójcik.

The results of the sensory evaluation of the low-carbohydrate bread enriched with pea protein powder are presented in Fig. 3.Sensory evaluation showed that the control bread, and the bread with PPP addition at the levels of 5, and 10% had the highest liking score. Similarly, Ziobro et al*.*^41^ demonstrated in the sensory evaluation of gluten-free bread with the addition of non-gluten proteins (i.e. albumin, soy, pea, lupine, collagen) that the bread supplemented with pea protein was the best assessed in case of structure, and porosity, and obtained the highest number of points for taste, and smell among the evaluated bread. The addition of higher levels of PPP (above 15%) caused an unpleasant aroma, and bitter taste. Furthermore, the low-carbohydrate bread generally received slightly low notes for the texture. It was caused by higher water addition, and the lack of gluten in the composition of flours used in the production of bread. It was also found that a higher percentage of pea protein caused crumbling of the crumb, and generally, the texture was not compact. The addition of PPP did not significantly change the bread crumb colour, which had a dark-brown colour that was generally acceptable for the evaluators.

### Amino-acid and fatty acid composition of low carbohydrate bread with optimal pea protein

Supplementation of low-carbohydrate bread with 10% of pea protein increased the content of all analyzed amino acids (Table 2). The chemical score (CS) for each essential amino acid has increased. The lowest value of CS was observed for leucine. EAAI value increased from 34 to 40 after the addition of the optimal protein supplement (10% PPP).

**Table 2:** Amino acid composition, basic chemical composition, and caloric value of control, and low-carbohydrate bread enriched with 10% of pea protein powder.

Amino acids	Amount of amino acid (mg∙g^−1^)	
	Control	10% of pea protein powder
Asparagine	32.1 ± 0.85*	36.5 ± 0.99**
Threonine	12.0 ± 0.25*	13.0 ± 0.30**
Serine	15.8 ± 0.71*	17.8 ± 0.68**
Glutamicacid	59.8 ± 1.39*	63.6 ± 1.10**
Proline	13.0 ± 0.37*	18.6 ± 0.38**
Glycine	15.2 ± 0.31*	16.1 ± 0.24*
Alanine	14.3 ± 0.46*	15.8 ± 0.61**
Cysteic acid	5.7 ± 0.23*	6.2 ± 0.30*
Valine	15.7 ± 0.44*	17.7 ± 0.46**
Methioninesulfone	5.5 ± 0.38*	5.73 ± 0.43*
Isoleucine	12.1 ± 0.31*	14.2 ± 0.39**
Leucine	20.5 ± 0.53*	23.3 ± 0.64**
Tyrosine	7.9 ± 0.19*	9.2 ± 0.21**
Phenylalanine	15.1 ± 0.41*	17.1 ± 0.48**
Histidine	7.4 ± 0.31*	8.4 ± 0.35*
Lysine	16.5 ± 0.42*	20.1 ± 0.41**
Arginine	26.1 ± 0.87*	31.3 ± 0.95**
Tryptophan	5.4 ± 0.32*	8.7 ± 0.41**
CS_Thre_(%)	30	33
CS_Val_(%)	31	35
CS_Met+Cyst_(%)	32	34
CS_Iso_(%)	30	36
CS_Leu_(%)	29	33
CS_Phe+Tyr_(%)	38	43
CS_Lys_(%)	30	37
CS_Tryp_(%)	54	87
EAAI (%)	34	40
**Basic chemical composition, and calorificvalue**		
Protein (%)	14.66 ± 0.43*	17.14 ± 0.58**
Carbohydrates (%)	18.38 ± 0.32*	16.94 ± 0.41**
Fiber (%)	14.55 ± 0.68*	13.74 ± 0.49*
Fat (%)	3.26 ± 0.15*	3.33 ± 0.19*
Calorific value (kcal/100 g)	191	194

Mean values followed by the same number of * within the same row are not significantly(α = 0.05) different.

According to Gorissen et al*.*^42^ pea as a plant-based protein source is rich in essential amino acids like lysine, and leucine, and non-essential amino acids like arginine, alanine, proline, and glutamic acid. The authors pointed out that pea has essential amino acid contents that meet the requirements as recommended by the WHO/FAO/UNU, and that the amount of essential amino acid in pea is higher than in plant materials, such as corn, soy, hemp, lupine, oat or brown rice. In the case of the content of glycine, cysteic acid, methionine sulfone, and histidine, only a slight increase in their content was detected in the composition of bread with 10% PPP addition, which was not statistically significant.

In the analyzed bread the following fatty acids were identified above 0.100 g/100 g: palmitic acid, octadecanoic acid, oleic acid + elaidic acid, linoleic acid + trans-9,12-octadecadienoic acid, and α-linolenic acid (Table 3). A significant increase of α-linolenic acid content in relation to the control bread occurred in the bread with 10% pea protein.

**Table 3:** Composition of fatty acids in the control bread, and bread with 10% of pea protein powder (PPP).

Fatty acids	Control Bread [g/100 g]	Bread with 10%PPP [g/100 g]
Caprylic acid (C8:0)	0.001 ± 0.000*	0.005 ± 0.000*
Capric acid (C10:0)	–	0.004 ± 0.000
Lauric acid (C12:0)	–	0.030 ± 0.002
Myristic acid (C14:0)	0.003 ± 0.000*	0.016 ± 0.001*
cis-9-Tetradecenoic acid (C14:ln5)	0.001 ± 0.000	–
Pentadecanoic acid (C15:0)	0.002 ± 0.000*	0.002 ± 0.000*
Palmitic acid (C16:0)	0.348 ± 0.004*	0.394 ± 0.004*
cis-9-Hexadecenoic acid (C16:ln7)	0.006 ± 0.000*	0.009 ± 0.000*
Heptadecanoic acid (C17:0)	0.009 ± 0.004*	0.003 ± 0.000*
cis-10-heptadecanoicacid (C17:ln7)	0.002 ± 0.000*	0.001 ± 0.000*
Octadecanoic acid (C18:0)	0.121 ± 0.007*	0.155 ± 0.004*
Oleic acid (C18:1n9c) + elaidic acid (C18:1n9t)	1.471 ± 0.003*	1.505 ± 0.005*
Linoleic acid (C18:2n6c) + trans-9,12-octadecadienoic acid (C18:2n6t)	1.218 ± 0.005*	1.010 ± 0.002*
α-Linolenic acid (C18:3n3(alpha))	0.952 ± 0.005*	1.364 ± 0.026**
Eicosanoic acid (C20:0)	–	0.024 ± 0.001
cis-11-Eicosenoic acid (C20:1n9)	0.031 ± 0.002*	0.037 ± 0.002*
cis-11,14-Eicosadienoic acid (C20:2n6)	0.002 ± 0.000*	0.003 ± 0.000*
Heneicosanoic acid (C21:0)	0.001 ± 0.000*	0.001 ± 0.000*
cis-11,14,17—Eicosatrienoic acid (C20:3n3)	–	0.001 ± 0.000
Behenic acid (C22:0)	0.018 ± 0.001*	0.024 ± 0.004*
Erucic acid (C22:1n9)	0.004 ± 0.000*	0.005 ± 0.003*
cis-13,16-Docosadienoic acid (C22:2n6)	–	0.001 ± 0.000
Tricosanoic acid (C23:0)	0.002 ± 0.000*	0.002 ± 0.000*
Lignoceric acid (C24:0)	0.012 ± 0.000*	0.016 ± 0.000*
cis-15-tetracosenoic acid (C24:1n9)	0.001 ± 0.000	–
SFA (Saturated fatty acid)	0.517 ± 0.022*	0.674 ± 0.018*
MUFA (Mono unsaturated fatty acid)	1.515 ± 0.034*	1.557 ± 0.041*
PUFA (Poly unsaturated fatty acid)	2.172 ± 0.050*	2.379 ± 0.058*
OMEGA 3	0.952 ± 0.037*	1.365 ± 0.049**
OMEGA 6	1.221 ± 0.051*	1.014 ± 0.067*
OMEGA 9	1.507 ± 0.048*	1.547 ± 0.050*

Mean values followed by the same number of * within the same row are not significantly (α = 0.05) different.

### Caloric value of low-carbohydrate bread with optimal pea protein amount

The addition of PPP had a significant effect on the chemical composition of the low-carbohydrate bread (Table 2). In the case of the bread with the optimum amount of pea protein at the level of 10% significant increases in the protein content (from 14.7% to 17.1%), and decrease in the carbohydrates content (from 18.4% to 16.9%) was noticed. According to García-Segovia^43^ the addition of 10% of pea protein increased protein content (to 19.3%), compared with the control wheat bread, and in the case of supplementation of wheat bread with pea protein concentrate (at the same level), as reported by Des Marchais^44^, even up to 20%. In other wheat bread studies, the protein content was 8.9%, carbohydrates were at 45.3% and after the addition of lupine isolate the protein content increased to almost 14.0%, and the carbohydrate content decreased to 37.9%^45^. The addition of faba bean flour (30%) wheat bread increased the protein content from 11.6 up to 16.5%^10^. The use of the addition of various types of lupine in the amount of 20% to wheat bread increased the protein content from 13.4% to about 19%, and the reduction of the carbohydrate content from 71% to about 60%. The fiber content increased from 9.2% to 15–16%^46^. In our study the fiber content of the protein-enriched bread was equal to 13.7%, there were no significant differences in the fiber, and fat content between the control bread, and the 10% PPP-based bread. The resulting low-carbohydrates, and high protein bread have a low-calorific value, compared to other breads whose calorific value in various publications were at a level of more than 200 kcal/100g^20,30^. Conventional wheat bread presents low protein, high carbohydrate, and small amounts of dietary fibre^6^. Our recipe, on the other hand, made it possible to obtain bread with increased protein content, and significantly reduced carbohydrate content.

## Conclusions

The pasting properties showed that mixing buckwheat flour with low-carbohydrate flaxseed flours significantly reduced peak viscosity, while technological additives (e.g. hydrocolloids, dietary fiber) significantly increased peak viscosity and reduced pasting temperature from 70 °C to 50 °C. The addition of pea protein (PPP) up to 10% did not significantly change pasting behavior, only higher amounts of proteins reduced viscosity parameters.

The results of volume, texture, and sensory evaluation indicated that enrichment of low-carbohydrate bread with pea protein powder addition up to 10% gave satisfactory results. However, the higher addition of this protein negatively influenced the volume, texture, and also taste, and odor of bread (crumbling of crumb, unpleasant aroma, and bitter taste). Pea protein-supplemented bread contained significantly higher amounts of amino acids (lysine, leucine, arginine, alanine, proline, glutamic acid, and tryptophan). The lowest value of chemical score was observed for leucine. EAAI value increased from 34 to 40 after the addition of the optimal protein supplement (10% PPP). Developed low-carbohydrate bread with increased protein was characterized by a carbohydrate content of 16.9%, protein content of 17.1%, the fiber content of 13.7%, and a calorific value of 194 kcal/100 g. This bread could be consumed for physically active people because of its role in the prevention of various human diseases. Besides, this kind of bread contains no gluten, and can be consumed by patients with celiac disease.

## Data Availability

All the data generated or analyzed during this study are included in this published article.
